# Scoping review of infectious disease prevention, mitigation and management in passenger ships and at ports: mapping the literature to develop comprehensive and effective public health measures

**DOI:** 10.1186/s41182-025-00681-0

**Published:** 2025-01-09

**Authors:** Lemonia Anagnostopoulos, Sotirios Vasileiadis, Leonidas Kourentis, Zacharoula Bogogiannidou, Ioanna Voulgaridi, Gordon Nichols, Fani Kalala, Matthaios Speletas, Christos Hadjichristodoulou, Varvara A. Mouchtouri, the EU HEALTHY SAILING project, Angelos Amditis, Spyridon Athanasiadis, Szava J. Bansaghi, Despoina A. Bygvraa, Stefanos Chatzimichelakis, Reuben D’Souza, Dimitra Dionysiou, Maria Guerrero-Vadillo, Giorgio Guzzetta, Volker Harth, Jan Heidrich, Jörn Klein, Jürgen F. Kolb, Prashant Kumar, Pierfrancesco Lepore, Sergiu Lupu, Valentina Marziano, Johannes Neumann, Symeon Nikolaou, Filip Nistor, Eleftherios Ouzounoglou, Vassilis Papataxiarhis, Marina Peñuelas, Patrizio Pezzotti, Catalin Popa, Raphael Rataj, Smaragda Reppa, Flavia Riccardo, Hannu Salmela, Niko Siilin, Konstantinos Theofilis, Constantinos Tsibanis, Carmen Varela, Nikolaos P. Ventikos, Georgios Vosinakis, Goran Vukelić, Christine Zädow, Vassilios Zagkas

**Affiliations:** 1 Laboratory of Hygiene and Epidemiology, Faculty of Medicine, University of Thessaly, 22 Papakyriazi Street, 41222 Larissa, Thessaly Greece; 2 HEALTHY SAILING Project, 22 Papakyriazi Street, 41222 Larissa, Thessaly Greece; 3 EU SHIPSAN Scientific Association, 22 Papakyriazi Street, 41222 Larissa, Thessaly Greece; 4 Department of Immunology & Histocompatibility, Faculty of Medicine, University of Thessaly, 41500 Larissa, Greece

**Keywords:** Infectious disease, Outbreak, Maritime, Passenger, Ship, Cruise, Ferry, Expedition, Port, Travel

## Abstract

**Background:**

With various infectious disease risks to passenger ship travellers, guidance for infectious disease prevention, mitigation and management (PMM) exists. Emerging infections and emergencies necessitate updated, context-specific guidelines and practices. New evidence for infection PMM must be translated into guidance for governmental authorities and the passenger ship industry. Under the European HEALTHY SAILING project, we conducted a scoping review of publications in PubMed, Scopus and grey literature for scientific articles, regulations, guidelines and policies describing infectious disease PMM in seaports, cruise, ferry, expedition and river cruise ships between 1990 and 2023.

**Main findings:**

Of 620 publications most were peer-reviewed articles (57.7%) and technical guidance (27.9%), followed by reports/other documents (9.1%), industry guidance (3.4%) and legislation (1.9%). Half (50.5%) of all publications addressed respiratory illnesses, fewer addressed gastroenteritis (11.5%), Legionnaire’s (6.1%), other vaccine-preventable (3.2%), vector-borne (1.6%) and sexually transmitted (1.0%) diseases. Most publications focus on infectious disease in seagoing cruise ships (75.7%) compared to ferries, expedition and river cruise ships (26.6%, 16.9%, 16.3%, respectively). Fewer publications addressed seaports (39.0%), shore-side personnel (19.7%) and port communities (2.4%). Most literature was published between 2020 and 2023 (50.2%) with a peak addressing respiratory illnesses (264 publications) during this period. A trend in volume and type was observed based on public health emergencies associated with the publication year.

**Conclusions:**

Peer-reviewed articles and guidance primarily address respiratory and gastrointestinal illnesses, seagoing cruise ships and onboard populations. Gaps on the following topics exist: other infectious disease types; other passenger ship types; land-based personnel and port communities. Future research could assess risk factors and PMM measure effectiveness considering vaccine-preventable, vector-borne and sexually transmitted diseases. The evidence-base should be strengthened to produce guidelines targeting specificities of seaports, ferries, expedition and river cruise ships. Developing guidelines to standardise passenger ship outbreak investigation reporting could help evaluate PMM measure effectiveness, the impact of passenger ship travel on port communities and vice versa. Modern passenger ship experiences—from educational to elderly focused cruising—present diverse public health risks, requiring continuous efforts by public health authorities and the shipping industry. While outside the review’s scope, measures may impact travellers’ mental health, necessitating strategies when designing and implementing PMM measures.

**Supplementary Information:**

The online version contains supplementary material available at 10.1186/s41182-025-00681-0.

## Background

Effective infectious disease prevention, mitigation and management (PMM) for passenger ship travel requires a multisectoral approach, with evidence-based protocols implemented by competent personnel from passenger shipping companies, governmental authorities and other relevant stakeholders. Evidence from infection prevention and control research in passenger ship settings must be translated into guidance, policies and regulations, culminating in evidence-based practices.

Local, regional and international level guidelines, policies and regulatory frameworks for infection PMM in passenger ships exist [1–4]. However, previously unknown emerging infectious diseases and new public health emergencies can impact passenger ship travel. Over the last decade, several public health emergencies of international concern (PHEIC) affected passenger shipping, including Ebola virus disease (2014–2016), Zika virus disease (2016), coronavirus disease (COVID-19) (2020–2023) and mpox previously known as monkeypox (2022–2023) [5–8]. Emerging infectious diseases and public health emergencies require up-to-date, context-specific guidelines and practices, integrating lessons from previous experiences. In response to emergencies, dedicated guidelines are developed considering specific pathogen characteristics, clinical features, epidemiology and transmission. Thus, research presenting new evidence on infectious disease PMM must be analysed to update and develop guidelines.

Infectious disease risks associated with passenger ship travel are well-recognised. Peer-reviewed publications describe outbreaks of norovirus, COVID-19, influenza, other vaccine-preventable and Legionnaire’s disease, with outbreaks facilitated by crowded environments, travellers from diverse origins, and common sources of potentially contaminated food and water [9–16]. Infection prevention and control guidelines are based on these publications; thus, it is critical to evaluate evidence assessing effectiveness of measures for infection PMM in specific contexts.

Multisectoral cooperation is essential for infectious disease preparedness and response, involving efforts of ship personnel, passenger ship companies and governmental authorities ashore [2]. Coordination and communication are required for interoperability of stakeholders’ preparedness plans, and to facilitate effective public health event management. International cooperation is also essential as passenger ships rapidly sail between countries, requiring coordination between countries and regions to promote common event management standards while avoiding inconsistent or contradictory responses [17]. Varied responses could lead to overreactions and possible international travel disruptions [18, 19].

Challenges faced by public health authorities and the passenger shipping industry during COVID-19 put renewed focus on the need for evidence-based practices to prevent and control infectious disease during passenger ship travel. The European HEALTHY SAILING project aims to introduce effective infectious disease PMM measures in large passenger ships [20]. This requires accumulating evidence for infection spread mechanisms on passenger ships, and building a “foundational knowledge base” to inform PMM measures development. A scoping review was conducted to first understand the extent of literature available on infectious disease PMM and passenger ship travel. Review results were created into a digital inventory, for use by HEALTHY SAILING as reference material when developing PMM measures. The scoping review attempted to answer the question: what is the published scientific literature, regulations, guidelines and policies about infectious disease PMM in large passenger ships and at seaports, worldwide? This paper provides a consolidated report compiling publications relevant to infectious disease PMM and passenger ship travel, considered valuable to the HEALTHY SAILING project, researchers, passenger shipping companies and relevant policymakers.

## Methods

A scoping review protocol was prepared and can be requested from the corresponding author. This review was reported in line with Preferred Reporting Items for Systematic Review and Meta-Analyses extension for Scoping Reviews (PRISMA-ScR) (Additional file 1 provides the completed PRISMA-ScR Checklist) [21].

### Eligibility criteria

Eligible publications were those addressing prevention, mitigation or management of any infectious disease type among human travellers linked to passenger ships or seaports. Publications were excluded if they referenced: seafarer health, shore-side workers or infectious disease frequency where ship type could not be determined; only vector surveillance at points of entry; historical maritime transport.

Passenger ships were those defined by the publication as a cruise ship, ferry, river cruise ship, or expedition-type ship. Expedition-type ships were defined as: “cruise ships” or “passenger vessels” or “expedition vessels” or “luxury vessels” sailing for extended periods in polar and/or remote regions, where medical support involved evacuation of many hours or days. Publications referring to mental health of passengers or crew members—even in the context of infectious disease—were considered outside the scope of the review. Publications referencing non-passenger ships (e.g., cargo/navy/research/migrant ships) were excluded. Grey literature referring generally to maritime travel, conveyances or points of entry were included.

No limitations were placed on geographic scope or publication type. English records published after 1990 were considered eligible and legislation/regulations enacted before 1990 but currently in force were included.

### Sources of information

PubMed and Scopus databases were searched in February 2023. Grey literature was searched among international/regional organisations [22–33], passenger ship industry and maritime health association webpages [34–39], and webpages of national-level maritime authorities, public health and transport agencies. Additional file 2 provides a complete list of information sources searched. The European EU HEALTHY GATEWAYS bibliography tool and a previous search conducted by GN were reviewed [40] and any eligible publications not identified through searches conducted in February 2023 were included.

### Search strategy and terms

Search terms were based on the literature and refined by the HEALTHY SAILING working group. Terms included variations of three concepts combined with Boolean operators: (i) disease; (ii) setting; and (iii) population.

### Study screening and selection

Records were imported into Endnote Library (version X7) with duplicates removed. Phase I screened titles and abstracts to ensure records were: published in English after 1990 (exception for legislation) and addressed infectious disease in human travellers. Records meeting phase I criteria continued to phase II full-text screening, to ensure they described infectious disease PMM linked to passenger ship travel or seaports. Two reviewers (LA, SV) divided phase I and II screening; uncertainties were verified by a third reviewer (VAM). Records meeting phase II criteria underwent data charting.

### Data charting

A data extraction template was prepared. Two reviewers (LA, SV) divided data charting; uncertainties were verified by a third reviewer (VAM). The following data were extracted from eligible records: (i) publication details; (ii) infectious disease type; (iii) maritime setting; (iv) population; (v) geographic region; and (vi) public health measures. Critical appraisal was not performed since the purpose was to understand the extent of existing literature, irrespective of its quality [21].

### Data synthesis

Records were categorised by specific variables they addressed (Table 1). Results were further analysed by publication year to identify publication trends.

**Table 1:** Categorisation of eligible records by publication type and other variables

Variables	Categorisations
Publication type	Peer-reviewed scientific article: original article, review or “other article” (e.g., commentary/editorial/case report)
	Technical guidance from international authorities or regional/national-level authorities
	Industry guidance at international or regional/national level
	Legislation/regulations at international or regional/national level
	Reports from international or regional/national-level authorities
	Other publications: press releases, leaflets, question & answer sheets, etc.
Infectious disease type	Respiratory illnesses: influenza, COVID-19, etc.
	Gastroenteritis: norovirus, salmonellosis, cyclosporiasis, etc.
	Legionnaire’s disease
	Other vaccine-preventable diseases (VPDs): varicella, measles, mumps, rubella, etc.
	Vector-borne diseases (VBDs): malaria, Zika virus disease, yellow fever, dengue, etc.
	Sexually transmitted infections (STIs): human immunodeficiency virus/acquired immunodeficiency syndrome (HIV/AIDS), mpox (previously known as monkeypox), etc.
	Other diseases: Ebola virus disease, Hepatitis E, filovirus, etc.
	Publications addressing more than one infectious disease type
Maritime setting	Publications address aspects onboard: cruise ships, passenger ferries, river cruise ships or expedition-type ships
	Publications address shore-side settings: seaports, land-based/port communities
Population	Ship-board populations: passengers and/or crew members
	Land-based shore-side/seaport personnel
	General public in seaport communities
Geographic region	Europe
	United States of America (USA)
	Other region
	Multiple regions
Public health measures	Measures applied to travellers: pre-embarkation screening, vaccination/prophylaxis, surveillance, diagnostic testing, isolation, treatment, contact tracing, quarantine, disembarkation of cases/contacts, education/training, non-pharmaceutical interventions
	Measures applied to inanimate objects: cleaning and disinfection, changes to ventilation operation, closure of onboard areas, discontinuation of activities, ship isolation/quarantine, inspection of conveyance/baggage/cargo

## Results

**Fig. 1:**
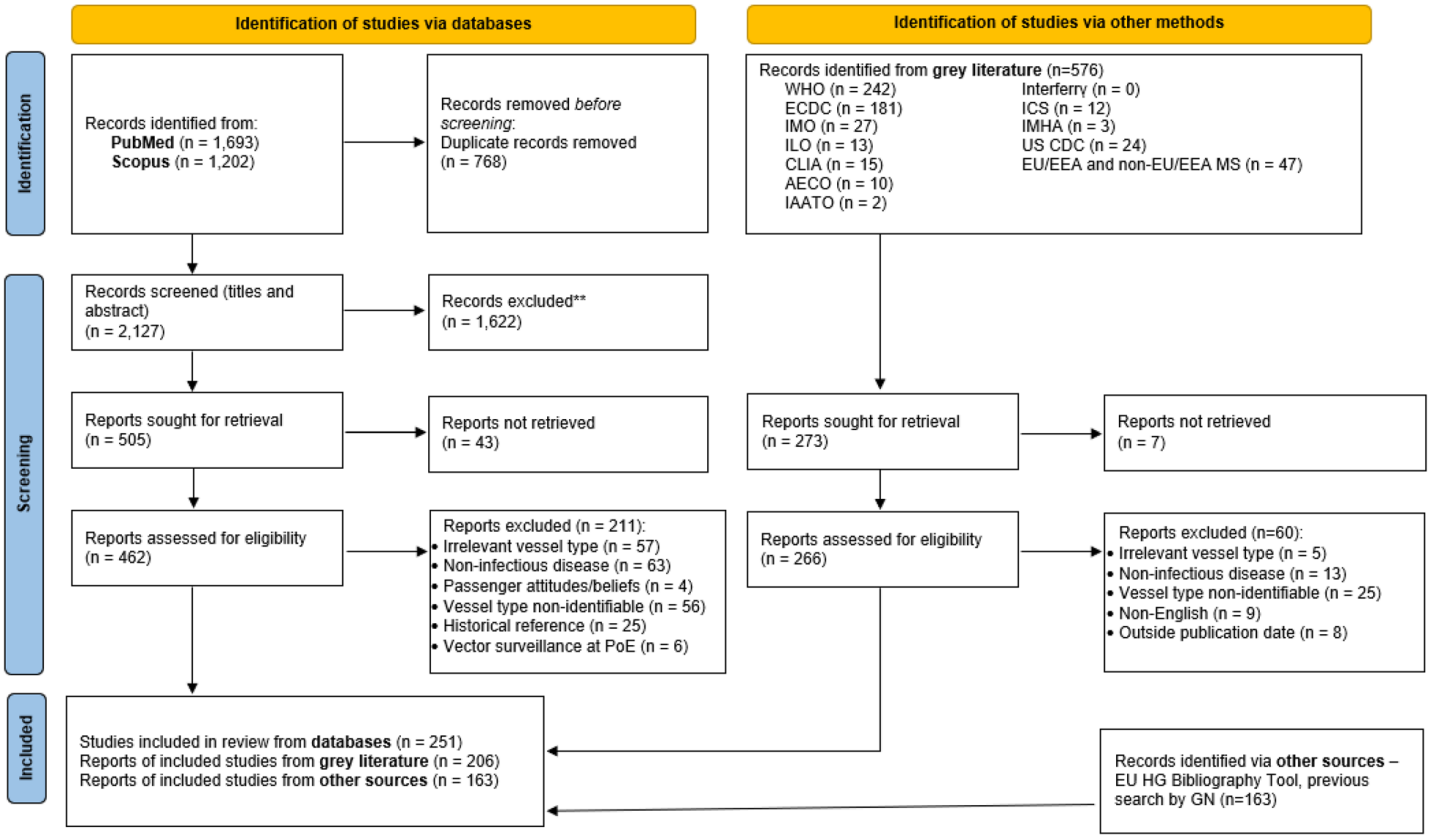
Flowchart of record selection for the scoping review

PubMed and Scopus searches identified 2,895 publications. Removing 768 duplicates, 2,127 records underwent phase I screening with 505 eligible for phase II (Fig. 1). In total, 462 database records underwent full-text screening, with 251 fulfilling eligibility criteria. Grey literature searches identified 576 records, with 206 meeting eligibility criteria. Searching other sources produced 163 eligible records. A total of 620 publications were included in this scoping review. Figure 1 describes reasons for excluding records. Given the vast number of eligible records, information for each publication including data extracted and links to each record (where available) are provided in Additional file 3.

Over half (57.7%) of eligible publications were peer-reviewed articles, followed by technical guidance documents (27.9%) (Table 2). Fewer publications were characterised as other records (4.7%), reports (4.4%), industry guidance (3.4%) and legislation/regulations (1.9%). Of 173 technical guidance documents, the majority (69.9%) were regional/national in nature, compared to 30.1% with an international focus.

**Table 2:** Number of included records, according to publication type and organisation

Publication type	Number of publications (% of 620)
Peer-reviewed scientific articles	358 (57.7%)
Original articles	236 (38.1%)
Other (editorials, viewpoints, case reports, etc.)	89 (14.4%)
Reviews	33 (5.3%)
Technical guidance documents	173 (27.9%)
World Health Organization (WHO)—headquarters	45 (7.3%)
World Health Organization (WHO)–regional offices	6 (1.0%)
International Maritime Organization (IMO)	4 (0.7%)
International Labour Organization (ILO)^a^	3 (0.5%)
European Centre for Disease Prevention and Control (ECDC)^b^	26 (4.0%)
European Commission	14 (2.3%)
EU Joint Action Healthy Gateways	30 (4.8%)
Other European level organizations / authorities	4 (0.7%)
European Union/European Economic Area Member States	12 (1.9%)
Centers for Disease Control and Prevention / Vessel Sanitation Program	19 (3.1%)
Other non-European Union / European Economic Area Member States	10 (1.6%)
Other^c^	29 (4.7%)
Reports^d^	27 (4.4%)
International focus	3 (0.5%)
Regional/national focus	24 (3.9%)
Shipping industry guidance	21 (3.4%)
Cruise Lines International Association	9 (1.5%)
International Chamber of Shipping	5 (0.8%)
International Maritime Health Association	1 (0.2%)
Association of Arctic Expedition Cruise Operators	3 (0.5%)
International Association of Antarctica Tour Operators	1 (0.2%)
American College of Emergency Physicians	1 (0.2%)
European Union / European Economic Area Member States	1 (0.2%)
Legislative/regulatory documents^d^	12 (1.9%)
International focus	7 (1.1%)
Regional/national focus	5 (0.8%)

^a^One publication issued jointly by ILO and IMO

^b^Two publications issued jointly by ECDC and European Maritime Safety Agency (EMSA)

^c^Other publications include press releases, online Q&As, communications from authorities, advice leaflets, etc. One publication considered as “other” is a pre-print article that had not undergone peer-review

^d^Reported by geographical level addressed

Table 3 shows the majority of grey literature originated from WHO (28.2%), followed by the European Joint Action HEALTHY GATEWAYS bibliography tool (23.7%).

**Table 3:** Number of eligible grey literature publications, according to source

Grey literature source	Number of publications (% of 262)
World Health Organization (WHO) (headquarters and regional offices)	74 (28.2%)
European Joint Action Healthy Gateways bibliography tool	62 (23.7%)
European Centre for Disease Prevention and Control (ECDC)^a^	36 (13.7%)
Centers for Disease Control and Prevention/Vessel Sanitation Program	22 (8.4%)
National authorities/agencies of European Union/European Economic Area Member States	14 (5.3%)
National authorities/agencies of non-European Union/European Economic Area Member States	17 (6.5%)
International Maritime Organization (IMO)	13 (5.0%)
Cruise Lines International Association	9 (3.4%)
International Chamber of Shipping	5 (1.9%)
International Labour Organization (ILO)^b^	5 (1.9%)
Association of Arctic Expedition Cruise Operators	3 (1.1%)
International Association of Antarctica Tour Operators	1 (0.4%)
International Maritime Health Association	1 (0.4%)

^a^Two publications issued jointly by ECDC and EMSA

^b^One publication issued jointly by ILO and IMO

Half of all publications addressed respiratory diseases (50.5%), with 270 (43.5%) specific to COVID-19. Fewer publications specifically addressed gastroenteritis (11.5%) and Legionnaire’s disease (6.1%), while a minimal number addressed other VPDs, VBDs and STIs (Table 4). An identical trend was observed among peer-reviewed scientific articles. Most technical guidance (56.6%) addressed respiratory diseases, however, a limited number focused solely on other infectious diseases. Nearly all industry-developed guidance focused on respiratory diseases (Table 4).

**Table 4:** Number of eligible records by infectious disease category, according to publication type

Infectious disease type	Number of publications			
	All publications (%)	Peer-reviewed scientific articles (%)	Technical guidance documents (%)	Industry guidance (%)
Respiratory diseases	313 (50.5%)	165 (46.1%)	98 (56.6%)	18 (85.7%)
Gastroenteritis	71 (11.5%)	65 (18.2%)	2 (1.2%)	1 (4.8%)
Legionnaire’s disease	38 (6.1%)	23 (6.4%)	9 (5.2%)	N/A
Other vaccine-preventable diseases	20 (3.2%)	17 (4.7%)	3 (1.7%)	N/A
Vector-borne diseases	10 (1.6%)	9 (2.5%)	1 (0.6%)	N/A
Sexually transmitted infections	6 (1.0%)	3 (0.85%)	3 (1.7%)	N/A
Other diseases ^a^	19 (3.1%)	4 (1.1%)	12 (7.0%)	N/A
More than one infectious disease type	143 (23.1%)	72 (20.1%)	45 (26.0%)	2 (9.5%)
Total	620 (100%)	358 (100%)	173 (100%)	21 (100%)

N/A: not applicable

^a^Other diseases include: Ebola virus disease, Hepatitis E, filovirus

As seen in Table 5, most publications addressed cruise ship settings and seaports (75.6% and 39.0%, respectively), regardless of publication type. Publications also focused on ship-board passenger and crew populations (64.2% and 56.9%, respectively), rather than land-based or general populations. Over half of publications addressed public health measures applied to travellers (57.1%), compared to only 37.4% referencing inanimate objects (Table 5).

**Table 5:** Number of eligible records according to maritime setting, population, geographic region and public health measures

Variable	Number of publications (% of 620)
Maritime setting^a^	
Cruise ship	469 (75.6%)
Passenger ferry	165 (26.6%)
Expedition-type vessel	105 (16.9%)
River cruise ship	101 (16.3%)
Seaport	242 (39.0%)
Port community	30 (4.8%)
Population^a^	
Passenger(s)	398 (64.2%)
Crew member(s)	353 (56.9%)
Land-based (shore-side / port personnel)	122 (19.7%)
General public (port community population)	15 (2.4%)
Geographic region	
Europe	190 (30.6%)
USA	55 (8.9%)
Other	147 (23.7%)
Multiple regions / all regions	226 (36.5%)
Unknown	2 (0.3%)
Public health measures addressed^a^	
Application of prevention, mitigation, management measures to travellers	354 (57.1%)
Application of prevention, mitigation, management measures to inanimate objects	232 (37.4%)

^a^When a single publication addressed more than one variable type, the publication was allocated to more than one variable

Table 6 demonstrates that application of PMM measures most often focused on cruise ships and seaports, with river cruise ships the least referenced context.

**Table 6:** Number of publications addressing public health measures, according to maritime setting

Maritime setting	Number of publications addressing public health measures^a^ (% of 620)	
	PMM travellers	PMM inanimate objects
Cruise ship	255 (41.1%)	195 (31.5%)
Passenger ferry	89 (14.4%)	86 (13.9%)
Expedition-type vessel	67 (10.8%)	50 (8.1%)
River cruise ship	63 (10.2%)	50 (8.1%)
Seaport	180 (29.0%)	106 (17.1%)
Port community	18 (2.9%)	11 (1.8%)

^a^PMM travellers: addresses application of public health measures to travellers; PMM inanimate objects: addresses application of public health measures to inanimate objects/environment

As seen in Table 7, nearly 40% of publications addressed respiratory disease on cruise ships, followed by those specific to gastroenteritis, Legionnaire’s disease and other VPDs in cruise ship settings (10.6%, 6.1%, 2.9%, respectively). Publications addressing infectious disease in the context of passenger ferries focused on respiratory diseases (10.0%), and to a lesser extent on Legionnaire’s disease (2.6%) and VBDs (0.8%). Limited publications were identified regarding infectious disease in expedition and river cruise settings, with the exception of respiratory diseases (7.7% and 6.9%, respectively). Publications specific to VBDs and STIs were minimal in any maritime setting (Table 7). A similar trend was observed among land-based settings, with most publications addressing seaports related to respiratory diseases (23.6%).

**Table 7:** Number of records per infectious disease type, by maritime setting, population and public health measures

Infectious disease type	Setting (% of 620)	Population^a^ (% of 620)	Application of public health measures^b^ (% of 620)	
			PMM travellers	PMM inanimate objects
Respiratory	Cruise: 234 (37.7%)	Passenger: 228 (36.8%)	219 (35.3%)	108 (17.4%)
	Ferry: 62 (10.0%)			
	Expedition: 48 (7.7%)	Crew: 208 (33.5%)		
	River cruise: 43 (6.9%)	Land-based: 77 (12.4%)		
	Seaport: 146 (23.5%)	General public: 10 (1.6%)		
	Port community: 19 (3.1%)			
Gastroenteritis	Cruise: 66 (10.6%)	Passenger: 60 (9.7%)	24 (3.9%)	37 (6.0%)
	Ferry: 3 (0.5%)			
	Expedition: 0 (0%)	Crew: 43 (6.9%)		
	River cruise: 4 (0.6%)	Land-based: 2 (0.3%)		
	Seaport: 2 (0.3%)	General public: 1 (0.2%)		
	Port community: 2 (0.3%)			
Legionnaire’s disease	Cruise: 38 (6.1%)	Passenger: 13 (2.1%)	4 (0.6%)	19 (3.1%)
	Ferry: 16 (2.6%)			
	Expedition: 2 (0.3%)	Crew: 6 (1.0%)		
	River cruise: 3 (0.5%)	Land-based: 0 (0%)		
	Seaport: 2 (0.3%)	General public: 0 (0%)		
	Port community: 0 (0%)			
Other vaccine-preventable diseases	Cruise: 18 (2.9%)	Passenger: 15 (2.4%)	17 (2.7%)	1 (0.2%)
	Ferry: 1 (0.2%)			
	Expedition: 0 (0%)	Crew: 16 (2.6%)		
	River cruise: 3 (0.5%)	Land-based: 3 (0.5%)		
	Seaport: 1 (0.2%)	General public: 3 (0.5%)		
	Port community: 2 (0.3%)			
Vector-borne diseases	Cruise: 4 (0.6%)	Passenger: 6 (1.0%)	6 (1.0%)	3 (0.5%)
	Ferry: 5 (0.8%)			
	Expedition: 2 (0.3%)	Crew: 1 (0.2%)		
	River cruise: 1 (0.2%)	Land-based: 0 (0%)		
	Seaport: 5 (0.8%)	General public: 0 (0%)		
	Port community: 0 (0%)			
Sexually transmitted infections	Cruise: 5 (0.8%)	Passenger: 3 (0.5%)	4 (0.6%)	2 (0.3%)
	Ferry: 0 (0%)			
	Expedition: 0 (0%)	Crew: 5 (0.8%)		
	River cruise: 1 (0.2%)	Land-based: 1 (0.2%)		
	Seaport: 1 (0.2%)	General public: 0 (0%)		
	Port community: 0 (0%)			
Other diseases^c^	Cruise: 8 (1.3%)	Passenger: 9 (1.5%)	16 (2.6%)	7 (1.1%)
	Ferry: 7 (1.1%)			
	Expedition: 7 (1.1%)	Crew: 7 (1.1%)		
	River cruise: 7 (1.1%)	Land-based: 7 (1.1%)		
	Seaport: 16 (2.6%)	General public: 0 (0%)		
	Port community: 0 (0%)			
More than one infectious disease type	Cruise: 96 (15.5%)	Passenger: 64 (10.3%)	64 (10.3%)	55 (8.9%)
	Ferry: 71 (11.5%)			
	Expedition: 46 (7.4%)	Crew: 67 (10.8%)		
	River cruise: 39 (6.3%)	Land-based: 32 (5.2%)		
	Seaport: 69 (11.1%)	General public: 1 (0.2%)		
	Port community: 7 (1.1%)			

^a^Passenger: on any passenger ship type; Crew: on any passenger ship type; Land-based: shore-side/port personnel; General public: port community populations

^b^PMM travellers: addresses application of public health measures to travellers; PMM inanimate objects: addresses application of public health measures to inanimate objects/environment

^c^Other diseases include: Ebola virus disease, Hepatitis E, filovirus

Most eligible records were published between 2020 and 2023 (50.2%), followed by 2015–2019 (13.7%) and 2010–2014 (12.3%) (Table 8). Peer-reviewed articles were most common across all years. Industry-produced guidance was only identified between 2020 and 2023. During each 5-year period between 1990 and 2009 and 2015–2019, the greatest proportion of publications were specific to gastroenteritis with the highest number (16 publications) during 2005–2009 (Table 8). After 2000, a greater volume of publications addressing respiratory diseases were identified, with an exponential increase in 2020–2023 (264 publications). The highest number of other VPD publications (nine) were observed during 2010–2014, while most VBD-specific publications were identified in 2015–2019 (malaria, Zika virus disease, Lyme disease). STI-specific publications peaked between 2020 and 2023 (primarily related to mpox), while other diseases were highest between 2010 and 2014 (11 publications primarily related to Ebola virus disease).

**Table 8:** Number of records per publication date, by publication type, disease, setting, population, region and measures

Publication year	Number of publications (% of 620)	Publication type: n (%)	Infectious disease type: n (%)	Maritime setting: n (%)	Population: n (%)	Geographic region: n (%)	Public health measures applied^a^: n (%)	
							PMM traveller	PMM object
1990–1994^b^	11 (1.8%)	Peer-reviewed: 5 (0.8%)	Gastroenteritis: 4 (0.6%)	Cruise: 11 (1.8%)	Passenger: 6 (1.0%)	USA: 3 (0.5%)	1 (0.2%)	3 (0.5%)
				Ferry: 6 (1.0%)				
		Legislation/ regulation: 4 (0.6%)	Legionnaire’s disease: 1 (0.2%)	Expedition: 6 (1.0%)	Crew: 10 (1.6%)	Multiple regions: 7 (1.1%)		
				River cruise: 6 (1.0%)				
		Technical guidance: 2 (0.3%)	More than one disease type: 6 (1.0%)	Seaport: 0 (0%)	Land-based: 0 (0%)	Other region: 1 (0.2%)		
				Port community: 0 (0%)	General public: 0 (0%)			
1995–1999	23 (3.7%)	Peer-reviewed: 22 (3.5%)	Respiratory: 3 (0.5%)	Cruise: 22 (3.5%)	Passenger: 22 (3.5%)	USA: 7 (1.1%)	8 (1.3%)	5 (0.8%)
			Gastroenteritis: 10 (1.6%)	Ferry: 2 (0.3%)				
			Legionnaire’s disease: 4 (0.6%)	Expedition: 0 (0%)	Crew: 13 (2.1%)	Europe: 4 (0.6%)		
		Technical guidance: 1 (0.2%)	Vaccine-preventable: 2 (0.3%)	River cruise: 0 (0%)	Land-based: 0 (0%)	Multiple regions: 10 (1.6%)		
			Vector-borne: 1 (0.2%)	Seaport: 1 (0.2%)	General public: 0 (0%)	Other region: 2 (0.3%)		
			More than one disease type: 3 (0.5%)	Port community: 0 (0%)				
2000–2004	38 (6.1%)	Peer-reviewed: 32 (5.2%)	Respiratory: 10 (1.6%)	Cruise: 34 (5.5%)	Passenger: 27 (4.4%)	USA: 5 (0.8%)	16 (2.6%)	15 (2.4%)
			Gastroenteritis: 13 (2.1%)	Ferry: 11 (1.8%)				
		Technical guidance: 4 (0.6%)	Legionnaire’s disease: 4 (0.6%)	Expedition: 4 (0.6%)	Crew: 26 (4.2%)	Europe: 6 (1.0%)		
		Legislation/ regulation: 1 (0.2%)	Vector-borne: 1 (0.2%)	River cruise: 4 (0.6%)	Land-based: 2 (0.3%)	Multiple regions: 19 (3.1%)		
		Report: 1 (0.2%)	Sexually transmitted: 1 (0.2%)	Seaport: 6 (1.0%)	General public: 0 (0%)	Other region: 8 (1.3%)		
			More than one disease type: 9 (1.5%)	Port community: 2 (0.3%)				
2005–2009	67 (10.8%)	Peer-reviewed: 46 (7.4%)	Respiratory: 6 (1.0%)	Cruise: 50 (8.1%)	Passenger: 37 (6.0%)	USA: 9 (1.5%)	26 (4.2%)	32 (5.2%)
		Technical guidance: 15 (2.4%)	Gastroenteritis: 16 (2.6%)	Ferry: 20 (3.2%)				
		Legislation/ regulation: 1 (0.2%)	Legionnaire’s disease: 14 (2.3%)	Expedition: 10 (1.6%)	Crew: 26 (4.2%)	Europe: 29 (4.7%)		
			Vaccine-preventable: 2 (0.3%)	River cruise: 13 (2.1%)	Land-based: 7 (1.1%)	Multiple regions: 23 (3.7%)		
		Report: 3 (0.5%)	Vector-borne: 2 (0.3%)	Seaport: 12 (1.9%)				
		Other: 2 (0.3%)	Other: 1 (0.2%)	Port community: 2 (0.3%)	General public: 1 (0.2%)	Other region: 6 (1.0%)		
			More than one disease type: 26 (4.2%)					
2010–2014	76 (12.3%)	Peer-reviewed: 46 (7.4%)	Respiratory: 12 (1.9%)	Cruise: 55 (8.9%)	Passenger: 42 (6.8%)	USA: 5 (0.8%)	54 (8.7%)	28 (4.5%)
		Technical guidance: 23 (3.7%)	Gastroenteritis: 8 (1.3%)	Ferry: 22 (3.5%)				
			Legionnaire’s disease: 2 (0.3%)		Crew: 40 (6.5%)	Europe: 23 (3.7%)		
		Legislation/ regulation: 1 (0.2%)	Vaccine-preventable: 9 (1.5%)	Expedition: 14 (2.3%)				
			Vector-borne: 1 (0.2%)		Land-based: 15 (2.4%)	Multiple regions: 37 (6.0%)		
		Report: 4 (0.6%)	Sexually transmitted: 1 (0.2%)	River cruise: 13 (2.1%)				
		Other: 2 (0.3%)	Other: 11 (1.8%)	Seaport: 33 (5.3%)	General public: 2 (0.3%)	Other region: 10 (1.6%)		
			More than one disease type: 32 (5.2%)	Port community: 2 (0.3%)				
2015–2019	85 (13.7%)	Peer-reviewed: 51 (8.2%)	Respiratory: 10 (1.6%)	Cruise: 61 (9.8%)	Passenger: 52 (8.4%)	USA: 14 (2.3%)	46 (7.4%)	29 (4.7%)
		Technical guidance: 24 (3.9%)	Gastroenteritis: 14 (2.3%)	Ferry: 26 (4.2%)	Crew: 40 (6.5%)	Europe: 22 (3.5%)		
		Legislation/ regulation: 2 (0.3%)	Legionnaire’s disease: 7 (1.1%)	Expedition: 17 (2.7%)				
		Report: 7 (1.1%)	Vaccine-preventable: 6 (1.0%)	River cruise: 13 (2.1%)	Land-based: 11 (1.8%)	Multiple regions: 33 (5.3%)		
		Other: 1 (0.2%)	Vector-borne: 5 (0.8%)	Seaport: 37 (6.0%)				
			Other: 7 (1.1%)	Port community: 2 (0.3%)	General public: 2 (0.3%)	Other region: 16 (2.6%)		
			More than one disease type: 36 (5.8%)					
2020–2023	311 (50.2%)	Peer-reviewed: 156 (25.2%)	Respiratory: 264 (42.6%)	Cruise: 228 (36.8%)	Passenger: 203 (32.7%)	USA: 12 (1.9%)	194 (31.3%)	115 (18.5%)
		Technical guidance: 104 (16.8%)	Gastroenteritis: 5 (0.8%)	Ferry: 77 (12.4%)				
		Industry guidance: 13 (2.1%)	Legionnaire’s disease: 6 (1.0%)	Expedition: 53 (8.5%)	Crew: 193 (31.1%)	Europe: 105 (16.9%)		
		Legislation/ regulation: 3 (0.5%)	Vaccine-preventable: 1 (0.2%)	River cruise: 49 (7.9%)	Land-based: 86 (13.9%)	Multiple regions: 89 (14.4%)		
		Report: 12 (1.9%)	Sexually transmitted: 4 (0.6%)	Seaport: 151 (24.4%)	General public: 10 (1.6%)	Other region: 104 (16.8%)		
		Other: 23 (3.7%)	More than one disease type: 31 (5.0%)	Port community: 22 (3.5%)		Unknown: 1 (0.2%)		
Unknown	9 (1.5%)	Industry guidance: 8 (1.3%)	Respiratory: 8 (1.3%)	Cruise: 8 (1.3%)	Passenger: 9 (1.5%)	Europe: 1 (0.2%)	9 (1.5%)	5 (0.8%)
				Ferry: 1 (0.2%)				
				Expedition: 1 (0.2%)	Crew: 5 (0.8%)			
		Other: 1 (0.2%)	Gastroenteritis: 1 (0.2%)	River cruise: 0 (0%)	Land-based: 1 (0.2%)	Multiple regions: 8 (1.3%)		
				Seaport: 2 (0.3%)	General public: 0 (0%)			
				Port community: 0 (0%)				

^a^PMM travellers: addresses application of public health measures to travellers; PMM inanimate objects: addresses application of public health measures to inanimate objects/environment

^b^Includes publications (legislation) published before 1990 but still in force

Cruise ships were the most commonly addressed setting across all years, with the number of publications increasing to 228 between 2020 and 2023. The volume of literature specific to expedition-style, river cruise ships and seaports progressively increased over the decades (Table 8). During 1990–1999, publications addressing regions/itineraries in the USA were greater than those addressing European regions/itineraries; this trend reversed during 2000–2004.

## Discussion

### Evidence by infectious disease

Regardless of publication type, our results reveal most publications focused on respiratory illnesses, with fewer publications addressing gastroenteritis and Legionnaire’s disease. Comparatively less publications exist specific to other VPDs, VBDs and STIs. This could indicate that while these diseases affect passengers and crew members they may occur less frequently, or it may reflect gaps regarding specific topics where evidence-based guidelines could be needed. Maritime health literature demonstrates respiratory illnesses including influenza and COVID-19 are prominent infections [9, 12, 15, 41, 42] with outbreaks reported on all passenger ship types [43–50]. The impact of gastroenteritis especially on cruise ships is also widely reported [10, 11, 51] through documented outbreaks [52–56]. However, other infectious diseases continue affecting passenger ship travellers. A systematic review identified outbreaks of VPDs in 13 cruise ships and one ferry [13]. The impact of VPDs extended to communities, as secondary measles cases were observed on land originating from a passenger ship traveller [13, 57]. Transport of VBD cases between geographic areas has been observed via passenger ferry travel [58, 59]. Moreover, a study among 21 passenger ferries identified infestations of flies, cockroaches, bedbugs and fleas [60]. Regarding STIs, a survey of seafarer sexual practices observed knowledge gaps that could increase infection risks; only about half of interviewed crew implemented safe practices with “occasional partners” [61].

The comparatively limited number of peer-reviewed articles addressing other VPDs, VBDs and STIs on passenger ships may indicate less research regarding risk factors for infection spread and effectiveness of PMM measures. This smaller evidence-base could challenge the development of effective guidelines, policies and practices. Expanding research for these diseases in different passenger ship types is essential to create evidence-based guidelines for public health authorities and the shipping industry. While there may be fewer publications specifically addressing the management of individual infectious diseases, global WHO and European guidance applying a generic approach on passenger ships is available [2, 3, 62, 63]. This “all-hazards” guidance for infectious diseases describes preparedness, case management, outbreak response and measures implementation.

Infectious disease cases and outbreaks associated with antibiotic resistant bacterial strains have been reported in cruise ship travellers [64, 65]. Considering the role of cross-border travel in the spread of antimicrobial resistant (AMR) bacteria, further research could investigate the influence of passenger ship travellers, aiming to mitigate the spread of AMR [66].

It is critical to also consider the impact of infectious disease events on the mental health of passengers and crew, since measures like isolation and quarantine can have negative effects. Studies assessing COVID-19 impacts found the pandemic posed a risk for specific psychological difficulties among the general Italian population, while quarantine was linked to increased stress, depression and post-traumatic stress disorder among Australian populations [67, 68]. Although these studies examined land-based populations, similar impacts may occur onboard. For crew particularly, this burden was evident early in the pandemic when crew changes and access to medical care ashore were challenged, contributing to stress, anxiety and depression [69]. When designing and implementing measures for infection PMM, it is essential to consider their potential impact and incorporate mental health policies and strategies.

### Evidence by maritime setting

Most literature concentrated on seagoing cruise ships; this focus could be due to cruise travel characterised by close contact interactions for extended periods, participation in land-based excursions and common food/water sources, factors which could increase infection risk [70]. Moreover, the global cruise sector is economically vital producing over $150 billion annually [71]. However, other passenger ship types provide significant economic and social contributions. The international ferry industry contributed $60 billion to the global GDP in 2019, with ferry lines having substantial economic and social impacts on remote areas providing connectivity, social services and essential goods [72].

Specificities of other passenger ship types may also facilitate infection introduction and spread. Ferries undertake short voyages with passengers widely dispersing at destinations. Infection may only be detected by land-based facilities days after passengers disembark, challenging effective PMM measures implementation. Outbreaks have been reported on passenger ferries [47, 73], and transport of malaria and tick-borne cases was observed between geographic areas with ferry transport [58, 59]. Expedition-type ships have limited access to medical facilities ashore and laboratory diagnostic capacities onboard, challenging effective PMM [74]. Infections have been reported among expedition ship travellers [74–76], and COVID-19 outbreaks in these settings occurred [46, 77, 78]. Respiratory illness and gastroenteritis outbreaks have also been reported among river cruise ships [79–83]. Smaller than seagoing cruise ships, they can carry several passengers participating in social activities such as dinners.

Guidelines and regulatory frameworks specifically addressing ferries, expedition-style and river cruise ships were surprisingly limited, considering outbreaks affect each setting. As specificities of each ship type impact infection spread and implementation of PMM measures, more context-specific guidelines for each setting are required. Additional research could assess risk factors and effectiveness of measures implemented during outbreaks, to inform evidence-based guidelines and policies.

Passenger ships are increasingly being used for diverse purposes, with the emergence of modern experiences such as multigenerational cruising, snowbird cruising among elderly populations and educational youth cruising [84–87]. Each experience presents varied public health risks; for example, risk factors and infection outcomes among young adults differ compared to older cruising populations. Continuous efforts from public health authorities and the shipping industry are required to meet diverse needs and risks presented by modern cruising experiences.

However, collecting infectious disease surveillance data to inform guidelines may be challenging, as access from passenger ship companies may be limited and certain ship types present additional barriers. Passenger ferries have shorter duration voyages and limited medical facilities/personnel onboard; infectious disease cases may be detected several hours or days after disembarkation in land-based facilities, making it difficult to link the event to passenger ship travel.

Fewer peer-reviewed articles and guidelines focused on seaports, indicating knowledge gaps about seaports’ roles in infection PMM and capacities they require. Attention was focused on COVID-19 PMM in seaports, specifically public health emergency contingency planning (PHECP) and implementation of International Health Regulations (IHR, 2005). While a positive outcome of the pandemic, this focus should extend beyond COVID-19, as seaports face other infectious disease threats and require capacities to respond to all event types. Guidance exists supporting seaport PHECP, however interactive and evidence-based tools for estimating seaport capacity needs would be valuable [88, 89].

### Evidence by population

Our results demonstrated that more publications addressed passengers compared to crew for nearly all infectious disease types, except other VPDs and STIs. While publications regarding occupational risks exist, crew spend extended periods onboard presenting more opportunities for exposure to possible risks, such as close contact interactions with infected persons. It is thus important to focus on the effectiveness of PMM measures considering occupational risks crew may face [90].

Limited literature focused on the impact of infection to shore-based personnel or community populations in the context of passenger ship travel. Both before and during COVID-19, secondary infectious disease cases affected land-based populations [48, 80, 91–93]. A systematic review reported over 260 secondary measles cases ashore linked to two ship outbreaks, and a respiratory outbreak identified four community cases of pandemic (H1N1) 2009 epidemiologically linked to cruise passengers [13, 48]. Conversely, cruise and expedition ship voyages often include land-based excursions; depending on the epidemiological situation ashore, these communities can be a risk factor for introducing infection onboard. Further retrospective studies are needed assessing the impact of passenger ship travel on land-based communities and vice versa. Current outbreak investigation reports in the literature are somewhat fragmented and may miss key information, including data to calculate attack rates, effectiveness of implemented response measures and epidemiologically linked community cases. Guidelines could be developed for reporting outbreak investigations on passenger ships, to standardise information collected by authorities. A “reporting template” would provide a more comprehensive understanding of outbreaks to evaluate the effectiveness of measures and the impact on land-based communities. This recommendation is supported in the literature; efforts are needed for research evaluating current measures and procedures for gathering and analysing outbreak data [94].

### Evidence by PMM measures

Whether analysed by type of ship or infectious disease, most publications focused on guidance or the application of PMM measures to travellers, compared to measures in the environment. Moreover, publications addressing PMM measures were predominantly available for cruise ships compared to other passenger ship types. This indicates less evidence and guidance for the effectiveness of PMM measures in passenger ferries, expedition-style and river cruise ships. A challenge for all passenger ship types is the systematic assessment of the most effective PMM measures. Outbreak investigations often report the implementation of multiple measures simultaneously, making it difficult to define the contribution of each individual measure. Moreover, outcomes in voyages immediately after a public health event are often unknown or unreported, making it difficult to evaluate the effectiveness of measures in halting an outbreak.

A promising measure for early detection of infectious diseases onboard is the use of wastewater monitoring. During the COVID-19 pandemic, studies on SARS-CoV-2 RNA wastewater surveillance among cruise ships concluded this could be a supplementary method for infectious disease surveillance [95, 96]. While further research is required, one event demonstrated that SARS-CoV-2 RNA was identified before confirmation of cases onboard, indicating the potential of waster surveillance to act as an early outbreak indicator [96].

### Publication trends

Assessing the literature in 5-year intervals from 1990, the number of publications steadily increased both in terms of peer-reviewed articles and technical guidance. A trend in volume and type was observed based on public health emergencies associated with the publication year. Between 2010 and 2014, the highest number of “other” publications addressed Ebola virus disease, corresponding to the 2014 WHO PHEIC declaration in response to the west African Ebola outbreak [97]. Most VBD publications were identified in 2015–2019, corresponding to WHO’s 2016 PHEIC declaration of Zika virus disease [98]. Publications related to STIs peaked in 2020–2023, correlating to the mpox outbreak and WHO’s 2022 PHEIC declaration [7]. An exponential increase in respiratory illness publications were observed during 2020–2023, coinciding with the COVID-19 pandemic. These findings may indicate that research, guidelines, policies and regulations are developed in response to public health threats either during or after the threat emerges, with less focus on preparedness for known or unknown infectious disease threats. Both during and after an event, it is essential to collect epidemiological information for understanding risk factors and documenting lessons for improving future response capacities. However, it is equally important to emphasise that evidence-based practices are in place before a threat emerges, to support preparedness capacities.

The scoping review found a nearly 30-fold increase in publications addressing respiratory illness on passenger ships during 2020–2023, compared to the previous 5-year period. Among peer-reviewed literature, most focused on COVID-19 outbreaks with several studies specific to the Diamond Princess cruise ship outbreak in February 2020. Considered one of the first COVID-19 outbreaks onboard a passenger ship, assessing response measures and outbreak dynamics helped establish an understanding of severe acute respiratory syndrome coronavirus (SARS-CoV-2) transmission, prevention and control. However, focusing research on only a few “monumental” events may miss lessons from outbreaks occurring in other regions, ship types, or during other periods of the pandemic. Furthermore, overpublishing research on certain infectious disease types may create gaps in evidence, guidance, regulations and practices.

Technical guidelines including from the European Centre for Disease Prevention and Control (ECDC), European Joint Actions and the Centers for Disease Control and Prevention (CDC) were largely available at regional or national level [99–101]. This could indicate a lack of harmonisation in guidelines and practices for infectious disease preparedness and response at global level. While it is necessary for guidance to be context-specific and compatible with the country and region, ships continuously sail between different countries and regions, necessitating multisectoral and international collaboration for infection PMM. Existing guidance from organisations such as WHO provide a global framework for regional and national authorities to align their guidance, promoting interoperable infection PMM practices between countries and regions. To continually improve global preparedness and response, these global frameworks may be updated considering lessons from recent or future public health emergencies. Any such updates would benefit from providing overarching global guidance for authorities to collect and record a standardised set of epidemiological information during passenger ship outbreak investigations.

The number of publications associated with the European region increased from six (2000–2004) to 29 (2005–2009). One possible explanation is the introduction of the European funded SHIPSAN project in 2006 addressing health, hygiene, infectious disease preparedness and response in maritime transport. Publications addressing the European region remained steady or increased after this, possibly explained by the continuation of European projects/joint actions with SHIPSAN TRAINET (2008–2011), SHIPSAN ACT (2013–2016) and EU HEALTHY GATEWAYS (2018–2022) [100, 102]. This highlights the importance of implementing and sustaining region-wide projects contributing to the scientific evidence-base, and developing guidance for infectious disease preparedness and response in passenger ships.

### Limitations

Given the volume of eligible publications, it was not feasible to perform critical appraisal. Thus, the strength of the evidence-base compiled cannot be guaranteed because the quality of literature is unknown. However, literature was identified from well-recognised and reputable sources including international/regional scientific agencies and governmental authorities. A limited amount of data was extracted and characterised for each publication. Nevertheless, the data collected fulfilled the review’s aims and will support the HEALTHY SAILING project. We also acknowledge that only literature available from sources relevant to the project, those published in English and after 1990 were eligible, leading to possible inclusion bias.

## Conclusions

This scoping review identified 620 publications addressing infectious disease PMM and passenger ship travel between 1990 and 2023. While the volume of publications steadily increased, gaps were identified. Literature primarily addresses respiratory illnesses, cruise ship settings and ship-board populations. Future research should focus on identifying risk factors and effectiveness of PMM measures considering VBDs, other VPDs and STIs. The evidence-base could be strengthened to produce guidelines for infection PMM targeting specificities of ferries, expedition-type ships, river cruise ships and seaports. Additional research is necessary to identify how land-based communities may introduce infection risk onboard, and how passenger ship travel impacts land-based communities. Furthermore, the development of guidelines for standardised outbreak investigation data collection and reporting on passenger ships could improve our understanding of the effectiveness of PMM measures, as well as impacts to and from land-based communities. The HEALTHY SAILING project conducted this scoping review to develop a digital inventory of publications [103]. Findings will be used by the project to conduct a systematic review considering for different passenger ships types the infectious disease frequency, burden of ship-borne disease to travellers and communities, risk factors for infection spread, and evidence for effectiveness of PMM measures.

## Supplementary Information

Additional file 1. Preferred Reporting Items for Systematic reviews and Meta-Analyses extension for Scoping Reviews Checklist. Provides the location of items reported in the manuscript in accordance with the PRISMA-ScR Checklist.

Additional file 2. Grey literature information sources used for scoping review. Provides a complete list of grey literature information sources searched for the scoping review.

Additional file 3. Data charting for scoping review of infectious disease PMM in passenger ships and ports—European HEALTHY SAILING project. Provides the data charted and URL link to the eligible records identified through the scoping review.

## Data Availability

The dataset supporting the conclusions of this article is available in the HEALTHY SAILING Scoping Search repository [https://healthysailing.eu/scoping-search-database-a/] and is also included in the article’s additional file (Additional file 3).

## Abbreviations

AMR: Antimicrobial resistance
CDC: Centers for Disease Control and Prevention
COVID-19: Coronavirus disease
ECDC: European Centre for Disease Prevention and Control
EMSA: European Maritime Safety Agency
HIV/AIDS: Human immunodeficiency virus/acquired immunodeficiency syndrome
IHR: International Health Regulations
ILO: International Labour Organization
IMO: International Maritime Organization
PHECP: Public health emergency contingency plan
PHEIC: Public health emergency of international concern
PMM: Prevention, mitigation and management
PRISMA-ScR: Preferred Reporting Items for Systematic Review and Meta-Analyses extension for Scoping Reviews
SARS-CoV-2: Severe acute respiratory syndrome coronavirus
STI: Sexually transmitted infection
USA: United States of America
VBD: Vector-borne disease
VPD: Vaccine-preventable disease
WHO: World Health Organization
