# Supplementary material for: Scoping review of infectious disease prevention, mitigation and management in passenger ships and at ports: mapping the literature to develop comprehensive and effective public health measures
Source: Trop Med Health. 2025 Jan 9;53:3. doi: 10.1186/s41182-025-00681-0 (PMC11716204; doi:10.1186/s41182-025-00681-0)
Supplement: Supplementary file 2 — Additional file 2. Grey literature information sources used for scoping review. Provides a complete list of grey literature information sources searched for the scoping review. [file 41182_2025_681_MOESM2_ESM.docx]

Grey literature information sources used for scoping review

The following tables present information sources used for the grey literature search.

**Table 1. Sources of information from international and regional organisations**

| International / regional organisation | Website (access date) |
| --- | --- |
| World Health Organization (WHO) headquarters | <https://iris.who.int/> (7 February 2023) |
| World Health Organization Regional Office for Africa (WHO AFRO) | <https://iris.who.int/> (8 February 2023) |
| World Health Organization Regional Office for the Americas (WHO AMRO) | <https://iris.who.int/> (8 February 2023) |
| World Health Organization Regional Office for South-East Asia (WHO SEARO) | <https://iris.who.int/> (7 February 2023) |
| World Health Organization Regional Office for Europe (WHO EURO) | <https://iris.who.int/> (7 February 2023) |
| World Health Organization Regional Office for the Eastern Mediterranean (WHO EMRO) | <https://iris.who.int/> (8 February 2023) |
| World Health Organization Regional Office for the Western Pacific (WHO WPRO) | <https://iris.who.int/> (8 February 2023) |
| European Centre for Disease Prevention and Control (ECDC) | <https://www.ecdc.europa.eu/en/search?s>=  (6 February 2023) |
| European Commission | <https://health.ec.europa.eu/index_en>  (23 February 2023) |
| International Labour Organization (ILO) | <https://www.ilo.org/global/publications/lang--en/index.htm>  (7 February 2023) |
| International Maritime Organization (IMO) | <https://www.imo.org/en/KnowledgeCentre/IndexofIMOResolutions/Pages/Default.aspx>  (10 February 2023) |
|  | <https://www.imo.org/en/publications/Pages/Home.aspx>  (10 February 2023) |

**Table 2. Sources of information from passenger ship industry and maritime health associations**

| Passenger ship industry / maritime health associations | Website (access date) |
| --- | --- |
| Cruise Lines International Association (CLIA) | <https://cruising.org/en/about-the-industry/policy-priorities/Public%20Health%20and%20Medical>  (17 February 2023) |
| Association of Arctic Expedition Cruise Operators (AECO) | <https://www.aeco.no/resources-and-tools/>  (17 February 2023) |
| International Association of Antarctica Tour Operators (IAATO) | <https://iaato.org/information-resources/data-statistics/iaato-atcm-information-papers/>  (10 February 2023) |
| Interferry | <https://interferry.com/overview/>  (10 February 2023) |
| International Chamber of Shipping (ICS) | <https://www.ics-shipping.org/resources/>  (17 February 2023) |
| International Maritime Health Association (IMHA) | <https://www.imha.net/>  (17 February 2023) |

**Table 3. Sources of information from EU/EEA MS and non-EU/EEA MS**

| Public health agencies, maritime authorities, transport agencies of EU/EEA and non-EU/EEA Member States | |
| --- | --- |
| Belgium | <https://www.sciensano.be>; https://www.health.belgium.be/fr |
| Bulgaria | <https://www.ncipd.org> |
| Croatia | <https://www.hzjz.hr> |
| Republic of Cyprus | <https://www.moh.gov.cy/moh/> |
| Czech Republic | <http://www.szu.cz> |
| Denmark | <https://www.sst.dk>; https://dma.dk/ |
| Estonia | <http://www.terviseamet.ee> |
| Finland | <https://thl.fi> |
| France | <http://www.santepubliquefrance.fr> |
| Germany | [https://www.rki.de](https://www.rki.de) |
| Greece | <https://eody.gov.gr/>; https://www.ynanp.gr/en/ |
| Ireland | <http://www.hpsc.ie> |
| Italy | <http://www.salute.gov.it> |
| Latvia | <https://www.spkc.gov.lv> |
| Lithuania | <http://sam.lrv.lt> |
| Malta | <https://deputyprimeminister.gov.mt> |
| Netherlands | <https://www.rivm.nl> |
| Poland | <http://www.pzh.gov.pl> |
| Portugal | <https://www.dgs.pt> |
| Romania | <http://www.insp.gov.ro> |
| Slovakia | <http://www.uvzsr.sk> |
| Slovenia | <http://www.nijz.si> |
| Spain | https://www.sanidad.gob.es/ |
| Sweden | <http://www.smittskyddsinstitutet.se> |
| United Kingdom | <https://www.gov.uk/government/organisations/uk-health-security-agency> |
| Iceland | <https://www.landlaeknir.is> |
| Norway | <https://www.fhi.no>; https://www.sdir.no/en/ |
| Centers for Disease Control and Prevention (US CDC) | <https://www.cdc.gov/> |
| Taiwan (R.O.C.) | <https://www.cdc.gov.tw> |
| China | <http://www.chinacdc.cn/en/> |
| Hong Kong | <https://www.chp.gov.hk> |
| Australia | <https://www.aihw.gov.au/>; https://www.health.nsw.gov.au/ |
| New Zealand | https://www.maritimenz.govt.nz/ |
| Japan | <https://www.niid.go.jp/niid/en/>; <https://www.niph.go.jp/index_en.html> |
| Brazil | <https://portal.fiocruz.br>; https://www.gov.br/anvisa/pt-br/english |
| Canada | <http://www.ciphi.ca>; <https://www.canada.ca/en/public-health.html>; https://tc.canada.ca/en |
